# Cannabinoid receptor 2 augments eosinophil responsiveness and aggravates allergen‐induced pulmonary inflammation in mice

**DOI:** 10.1111/all.12858

**Published:** 2016-03-16

**Authors:** R. B. Frei, P. Luschnig, G. P. Parzmair, M. Peinhaupt, S. Schranz, A. Fauland, C. E. Wheelock, A. Heinemann, E. M. Sturm

**Affiliations:** ^1^ Institute of Experimental and Clinical PharmacologyMedical University of GrazGrazAustria; ^2^ Division of Physiological Chemistry IIDepartment of Medical Biochemistry and BiophysicsKarolinska InstitutetStockholmSweden

**Keywords:** airway hyperresponsiveness, Cannabinoid receptor 2, Eosinophils, Ovalbumin‐induced asthma, Priming

## Abstract

**Background:**

Accumulation of activated eosinophils in tissue is a hallmark of allergic inflammation. The endocannabinoid 2‐arachidonoylglycerol (2‐AG) has been proposed to elicit eosinophil migration in a CB_2_ receptor/G_i/o_‐dependent manner. However, it has been claimed recently that this process may also involve other mechanisms such as cytokine priming and the metabolism of 2‐AG into eicosanoids. Here, we explored the direct contribution of specific CB_2_ receptor activation to human and mouse eosinophil effector function *in vitro* and *in vivo*.

**Methods:**

*In vitro* studies including CB_2_ expression, adhesion and migratory responsiveness, respiratory burst, degranulation, and calcium mobilization were conducted in human peripheral blood eosinophils and mouse bone marrow‐derived eosinophils. Allergic airway inflammation was assessed in mouse models of acute OVA‐induced asthma and directed eosinophil migration.

**Results:**

CB_2_ expression was significantly higher in eosinophils from symptomatic allergic donors. The selective CB_2_ receptor agonist JWH‐133 induced a moderate migratory response in eosinophils. However, short‐term exposure to JWH‐133 potently enhanced chemoattractant‐induced eosinophil shape change, chemotaxis, CD11b surface expression, and adhesion as well as production of reactive oxygen species. Receptor specificity of the observed effects was confirmed in eosinophils from CB_2_ knockout mice and by using the selective CB_2_ antagonist SR144528. Of note, systemic application of JWH‐133 clearly primed eosinophil‐directed migration *in vivo* and aggravated both AHR and eosinophil influx into the airways in a CB_2_‐specific manner. This effect was completely absent in eosinophil‐deficient ∆dblGATA mice.

**Conclusion:**

Our data indicate that CB_2_ may directly contribute to the pathogenesis of eosinophil‐driven diseases. Moreover, we provide new insights into the molecular mechanisms underlying the CB_2_‐mediated priming of eosinophils. Hence, antagonism of CB_2_ receptors may represent a novel pharmacological approach for the treatment of allergic inflammation and other eosinophilic disorders.

**Abbreviations**
2‐AG: 2‐arachidonoylglycerol
Ab: antibody
AF: Alexa Fluor
AHR: airway hyperresponsiveness
bmEos: mouse bone marrow‐derived eosinophils
CI: chemotactic or chemokinetic index
Cy5: cyanine 5
CysLTs: cysteinyl leukotrienes
DCF: 2′7′‐dichlorofluorescein
DCFDA: 2′,7′‐dichlorofluorescin diacetate
ERK: extracellular signal‐regulated kinase
FSC: forward scatter
IL: interleukin
LC‐MS: liquid chromatography–mass spectrometry
LT(x): leukotriene
MAPK: mitogen‐activated protein kinase
MEK: mitogen/extracellular signal‐regulated kinase
OVA: ovalbumin
PAF: platelet‐activating factor
PBMC: peripheral blood mononuclear cells
PE: phycoerythrin
PG: prostaglandin
PI3K: phosphoinositide 3‐kinase
PMNL: polymorphonuclear leukocytes
PTX: pertussis toxin
ROCK: rho‐associated protein kinase
SSC: side scatter

Eosinophils are potent effector cells in the pathogenesis of many disorders, ranging from allergy and bronchial asthma 1, to eosinophilic esophagitis 2, colitis ulcerosa 3, hypereosinophilic syndrome 4, and renal disease 5. Activated eosinophils are one of the primary sources of cytotoxic proteins, proinflammatory cytokines, and growth factors, such as IL‐4, IL‐5, IL‐10, and IL‐13 6, and promote numerous immunoregulatory functions that lead to the progression of inflammation, mucus secretion, tissue remodeling, and angiogenesis 7.

Regarding asthma, levels of eosinophil granule proteins such as major basic protein (MBP) or eosinophil peroxidase (EPO) broadly correlate with disease severity 8. Moreover, it has been shown that patients who receive treatment based on eosinophil counts in sputum have significantly fewer severe asthma exacerbations than patients treated according to standard management therapy 9.

Corticosteroids are currently the most effective treatment to reduce eosinophil numbers in the blood and tissue. However, the pleiotropic effects, especially of orally applied corticosteroids to control severe asthma, can result in potentially harmful side‐effects and thus limit their therapeutic use 10. Therefore, considerable effort has been invested in the development of drugs that can effectively control the trafficking and activation of eosinophils to ameliorate the inflammatory response. Current clinical trials with the eosinophil‐targeted mepolizumab revealed an oral glucocorticoid‐sparing effect and significantly reduced exacerbation rates in well‐selected patients with persistent eosinophilic asthma; however, FEV_1_ seemed to remain largely unaffected 11, 12. Of note, asthma is a heterogeneous condition with diverse characteristics and disease variants. Thus, stratification of patients by clinical characteristics (phenotypes) and pathogenetic mechanisms (endotypes) should lead to more targeted and personalized approaches to asthma therapy. Endocannabinoids are bioactive lipids released from the cell membrane upon cell activation. The two main endocannabinoids are the arachidonic acid (AA) derivatives 2‐arachidonoylglycerol (2‐AG) 13 and *N*‐arachidonoylethanolamine (anandamide (AEA)) 14. Most of their actions are mediated by two G‐protein‐coupled receptors, the cannabinoid receptors 1 and 2 (CB_1_ and CB_2_). In contrast to CB_1_, which is abundantly expressed in the nervous system 15, CB_2_ is mainly found in lymphoid organs and cells of the immune system 16. Among others, CB_2_ expression has been reported for B cells, monocytes/macrophages, and eosinophils, indicating a crucial immunoregulatory role for CB_2_ and its ligands 17.

Up to now, only three studies have examined the direct contribution of the endocannabinoid 2‐AG on eosinophil migration *in vitro*. Oka et al. demonstrated that 2‐AG induces the chemotaxis of EoL‐1 cells and human peripheral blood eosinophils in a CB_2_‐dependent manner, although the order of the pharmacologically effective concentration of 2‐AG was significantly higher than that of other chemoattractants 18. Interestingly, another group confirmed this minimal CB_2_‐mediated effect of 2‐AG and suggested that 2‐AG‐induced migration is a complex process that may involve other mechanisms, such as cytokine priming, rapid 2‐AG metabolism into eicosanoids, and further generation of 15‐lipoxygenase metabolites 19.

Moreover, due to the use of different rodent models and varying pharmacological approaches, the *in vivo* role of CB_2_ in eosinophilic disorders is still uncertain. Indeed, a CB_2_ inverse agonist was shown to block ovalbumin (OVA)‐induced lung eosinophilia in mice 20. Accordingly, the CB_2_ antagonist SR144528 mediated beneficial effects in oxazolone‐induced contact dermatitis 21. In contrast, Giannini et al. evaluated the effects of the CB_1_/CB_2_ receptor agonist CP55,940 on OVA‐induced asthma in guinea pigs and concluded that both CB_1_ and CB_2_ receptors are involved in lung protection 22. Similarly, THC reduced cytokine and IgE level as well as mucus production in OVA‐challenged mice 23; however, the use of CB_1_/CB_2_ knockout mice revealed that the effects of THC were cannabinoid receptor‐independent 24.

Because of the conflicting reports on the pathophysiological role of CB_2_ receptors, we set out to explore the direct contribution of specific CB_2_ activation on eosinophil effector function. To this end, the effects of the endocannabinoid 2‐AG and the potent and selective CB_2_ receptor agonist JWH‐133 were studied *in vitro* and in mouse models of acute OVA‐induced asthma and directed eosinophil migration. Our results provide clear evidence for a novel CB_2_‐induced eosinophil‐specific ‘priming mechanism’ that potentiates eosinophil effector function *in vitro* and enhances bronchial inflammation *in vivo*, reflected by both increased airway resistance and eosinophil influx into the airways.

## Materials and methods

Detailed materials and methods are provided in the supplement.

### Preparation of mouse eosinophils

Bone marrow‐derived eosinophils (bmEos) were differentiated *ex vivo* from unselected bone marrow progenitors using a well‐defined cytokine regimen 25.

### Flow cytometric staining of CB_2_ receptors on eosinophils

Purified human eosinophils were stained with a polyclonal rabbit anti‐human CB_2_ primary Ab or isotype control, followed by a goat anti‐rabbit secondary Ab (AF‐647). CB_2_ expression was quantified by flow cytometry.

### Shape change assay

Isolated eosinophils, PMNL or PBMC were pretreated as indicated and stimulated. Shape change was estimated by flow cytometry as the increase of forward scatter 26, 27.

### Migration

Chemotaxis and Chemokinesis: Purified human eosinophils or bmEos were pretreated as indicated, placed into the top of a 48‐well micro‐Boyden chamber (human) or a 96‐well chemotaxis plate (bmEos), and were allowed to migrate toward the indicated chemoattractant or vehicle.

### Calcium flux

Isolated human eosinophils or bmEos were loaded with Fluo‐3‐AM in the presence of 0.02% pluronic F‐127 28. Changes in intracellular Ca^2+^ were detected as fluorescence increase in the FL1‐(530/30 nm) channel.

### Adhesion assay under flow

Vena8^™^ biochips (Cellix Ltd., Dublin, Ireland) were coated with ICAM‐1, and coated channels were superfused with purified eosinophils. Adhesion was monitored using a Hamamatsu ORCA‐03G digital camera and CellixVenaFlux software.

### CD11b‐upregulation

Whole blood samples or PMNL were pretreated as indicated and were incubated with agonists for 30 min at 37°C 29. Samples were stained with anti‐CD16‐PE‐Cy5 and anti‐CD11b‐PE (ICRF44) Ab. CD11b upregulation was analyzed by flow cytometry.

### *In vivo* chemotaxis

Eight‐week‐old IL‐5Tg mice were treated i.p. with JWH‐133 (5 mg/kg/day) or vehicle for three consecutive days. *In vivo* chemotaxis of eosinophils was induced by intranasal instillation of 4 μg eotaxin‐2/CCL24. Bronchoalveolar lavage fluid (BALF) was collected 5 h postinstillation, and migration was evaluated by flow cytometric counting of highly granular (high side scatter) CD11c^−^/Siglec F^+^ cells.

### Mouse model of allergic lung inflammation

Eight‐week‐old female C57Bl6/N mice were immunized by i.p. injections of 10 μg of OVA adsorbed to Al(OH)_3_ on days 0 and 7. Mice were challenged by an aerosol of OVA in saline on days 14 and 16. Additionally, mice received a daily i.p. injection of CB_2_ agonist/antagonist (10 mg/kg) or vehicle on day 9 to day 16. On day 17, either airway hyperresponsiveness to methacholine was recorded with the FlexiVent system (Scireq, Montreal, QC, Canada) or BALF was taken and analyzed by flow cytometry.

## Results

### CB_2_ receptor expression is enhanced on eosinophils from allergic donors

**Figure 1:**
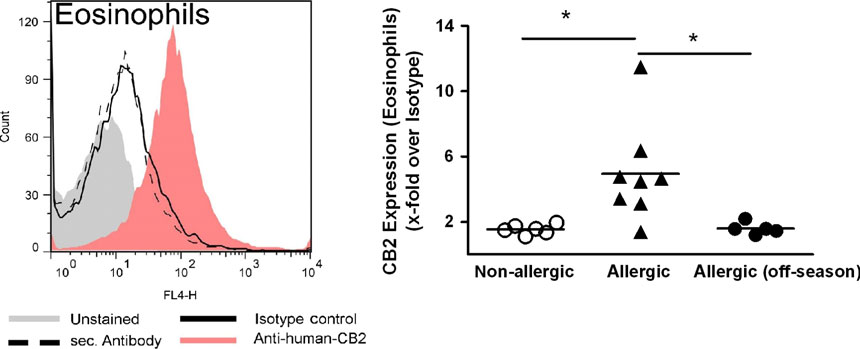
CB_2_ expression is enhanced on eosinophils from symptomatic allergic donors. Purified human eosinophils were stained with a polyclonal rabbit anti‐human CB_2_ primary Ab or isotype control (1 : 50), followed by a goat anti‐rabbit secondary Ab (AF‐647; 1 : 500).

CB_2_ expression has been reported for B cells, monocytes/macrophages, NK cells, basophils, and eosinophils, and at lower levels, for neutrophils and T cells 16, 30, 31. In this study, CB_2_ surface expression was confirmed by flow cytometric staining in‐ and off‐season in human peripheral blood eosinophils from allergic volunteers with seasonal respiratory symptoms and healthy subjects. Of note, quantification revealed ~3.4‐fold higher expression of CB_2_ on eosinophils from symptomatic allergic donors compared to healthy controls. Interestingly no CB_2_ upregulation was observed off‐season in eosinophils from asymptomatic allergic donors (Fig. 1).

### The CB_2_ receptor agonist JWH‐133 specifically enhances human and mouse eosinophil function

**Figure 2:**
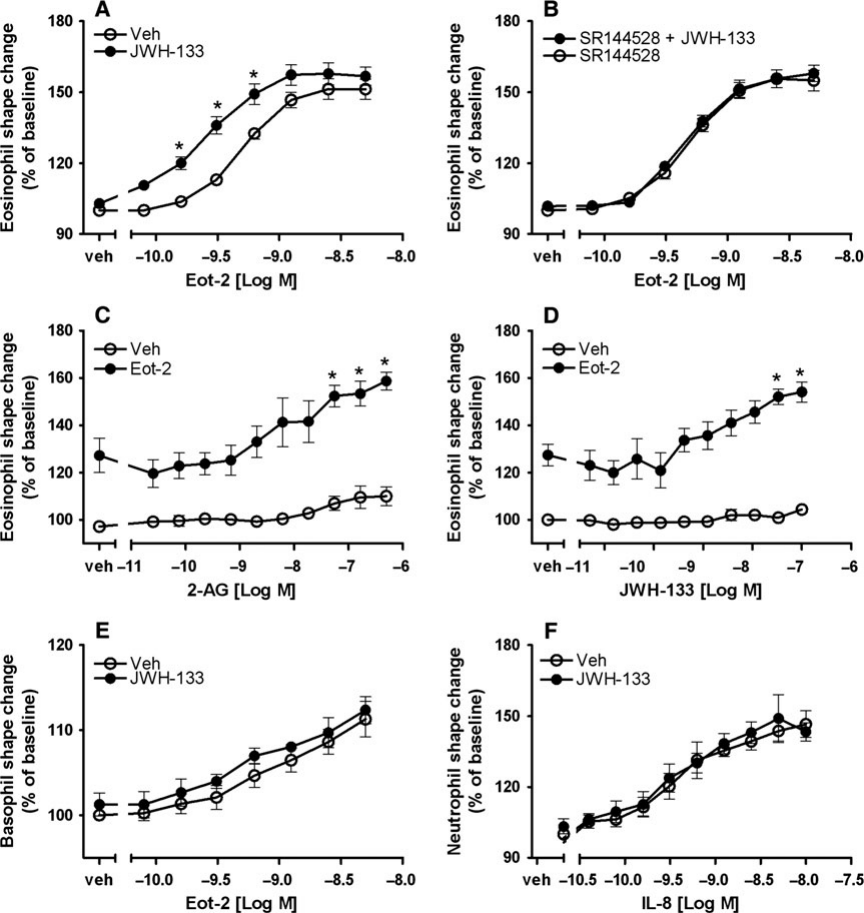
JWH‐133 differentially modulates shape change in human eosinophils, basophils and neutrophils. (A) Eosinophils were pretreated with JWH‐133 (100 nM) or vehicle and stimulated with eotaxin‐2/CCL24 (Eot‐2). (B) Prior to JWH‐133 treatment, eosinophils were incubated with SR144528 (1 μM) and then treated as described in (A). (C and D) Eosinophils were pretreated with 2‐AG or JWH‐133 and stimulated with eotaxin‐2/CCL24 (0.6 nM) or vehicle. (E) Basophils (HLA‐DR ^−^/CD123^+^ PBMC) were treated as described in (A). (F) Neutrophils (CD16^+^ PMNL) were pretreated with JWH‐133 (100 nM) or vehicle and stimulated with IL‐8. Data are shown as mean ± SEM. *P < 0.05 *vs* vehicle *n* = 4–7.

Conflicting data exist regarding the effect of CB_2_ activation in eosinophils and neutrophils, and little is known about its impact on basophil function 32, 33. Thus, here we explored the effect of the selective CB_2_ agonist JWH‐133 on human eosinophils, neutrophils, and basophils by means of shape change. Having encountered a chemotactic factor *in vivo*, leukocytes immediately begin to rearrange their cytoskeleton and change their shape to facilitate their attachment to microvascular endothelial cells. Such morphological changes can be detected by flow cytometry as changes in the forward scatter properties of the cells 34. Representative plots are provided in Fig. S5. First, purified eosinophils were pretreated with JWH‐133 (100 nM) or vehicle, cells were stimulated with serial dilutions of eotaxin‐2/CCL24, and shape change was assessed by flow cytometry. As shown in Fig. 2A, JWH‐133 led to a significant increase of eosinophil responses; particularly, the sensitivity to eotaxin‐2/CCL24 was increased up to threefold. To verify that this effect was mediated through selective CB_2_ activation, eosinophils were exposed to the CB_2_ antagonist SR144528 (1 μM, 10 min at RT) prior to the assay. As illustrated in Fig. 2B, SR144528 totally prevented the enhancing effect of JWH‐133. Similarly, the endocannabinoid 2‐AG concentration‐dependently increased eotaxin‐2/CCL24‐induced shape change and elicited a weak, but dose‐dependent shape change by itself with a maximum response observed at 500 nM (Fig. 2C), similar to JWH‐133 (Fig. 2D). Emphasizing the cell type‐specific impact of selective CB_2_ activation, pretreatment with JWH‐133 only slightly enhanced basophil shape change as induced by eotaxin‐2/CCL24 (Fig. 2E) and did not affect neutrophil responses to IL‐8 (Fig. 2F).

**Figure 3:**
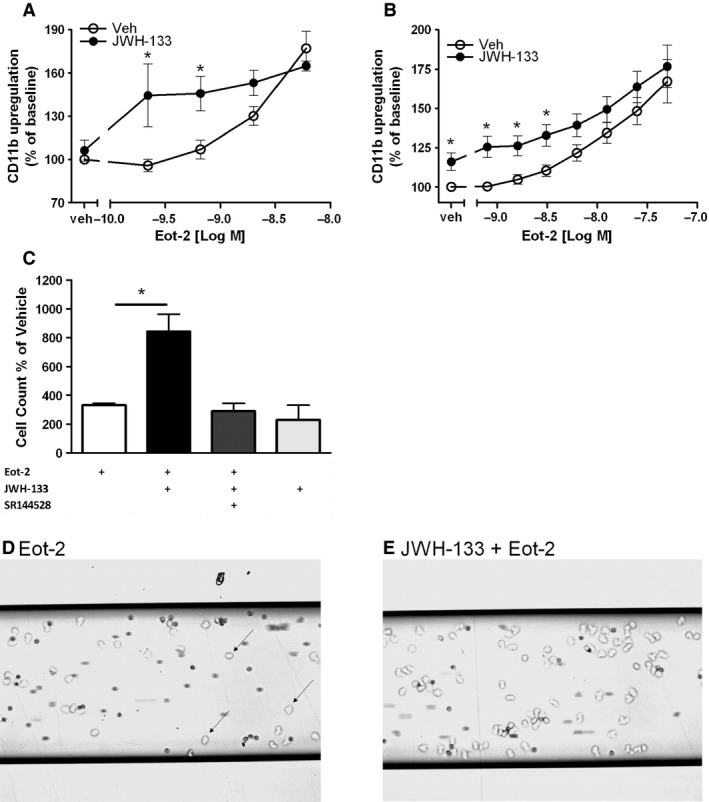
JWH‐133 primes human and mouse eosinophils for an enhanced CD11b expression and adhesion. (A) Human whole blood samples were stained with anti‐CD11b‐PE and anti‐CD16‐PE‐Cy5 Ab and pretreated with JWH‐133 (1 μM) or vehicle and then stimulated with eotaxin‐2/CCL24. (B) bmEos were stained with anti‐mouse CD11b‐PE Ab, pretreated with JWH‐133 (250 nM) or vehicle and stimulated with eotaxin‐2/CCL24. (C, D and E) Eosinophils were pretreated with SR144528 (1 μM) or vehicle, treated with JWH‐133 (250 nM) or vehicle, and stimulated with eotaxin‐2/CCL24 (0.6 nM). ICAM‐1‐coated channels were superfused with human eosinophils, and tightly adherent eosinophils (black arrows) were counted. Data are shown as mean ± SEM, *P < 0.05 *n* = 4–8.

Besides shape change, the chemoattractant‐induced upregulation of adhesion molecules such as α_m_β_2_ integrins (CD11b/CD18; Mac‐1) is another prerequisite for eosinophil effector functions 35. Thus, human eosinophils in whole blood and mouse bmEos were pretreated with JWH‐133 (1 μM and 250 nM, respectively) or vehicle and stimulated with eotaxin‐2/CCL24. Interestingly, JWH‐133 clearly potentiated the ability of eotaxin‐2/CCL24 to upregulate CD11b in human eosinophils, whereas in mouse eosinophils, JWH‐133 directly induced CD11b surface expression. At lower concentrations of eotaxin‐2/CCL24, JWH‐133 led to a ~50% increase of CD11b in human eosinophils (Fig. 3A) and to a ~25% increase in bmEos (Fig. 3B). Similar modulatory effects were observed with 2‐AG (250 nM) (Fig. S1).

The same pattern of CB_2_‐mediated priming was observed in adhesion assays under flow conditions. Pretreatment of purified human eosinophils with 100 nM JWH‐133 led to a significant twofold increase of firm adherent cells on ICAM‐1 coated channels compared to eotaxin‐2/CCL24 alone. Again, this effect was completely prevented with the CB_2_ antagonist SR144528 (1 μM) (Fig. 3C). Representative pictures are provided in Fig. 3D and E.

**Figure 4:**
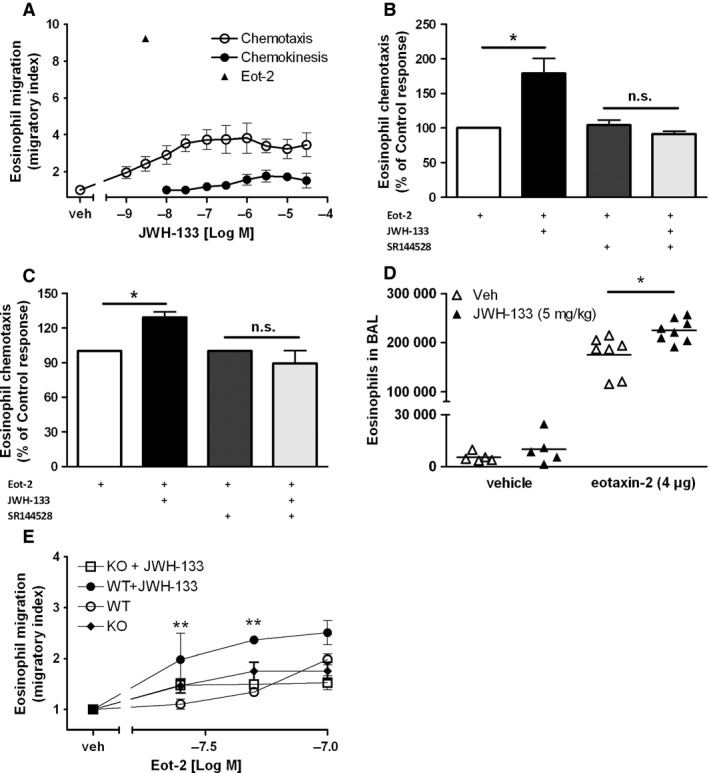
JWH‐133 enhances the migratory responsiveness of human and mouse eosinophils. (A) Chemotaxis: eosinophils were allowed to migrate toward serial dilutions of JWH‐133, vehicle, or eotaxin‐2/CCL24. Chemokinesis: Eosinophils were pretreated with serial dilutions of JWH‐133 or vehicle and were allowed to migrate toward assay buffer only. (B) eosinophils were pretreated with JWH‐133 (5 nM) and were allowed to migrate toward eotaxin‐2 (1 nM). (C) bmEos were pretreated with vehicle or SR144528 (1 μM), mixed with JWH‐133 (250 nM) or vehicle, and cells were allowed to migrate toward eotaxin‐2/CCL24 (100 nM). (D) IL‐5Tg BALB/c mice were treated i.p. with JWH‐133 (5mg/kg/d) or vehicle for 3 days. Five hours after intranasal application of 4 μg eotaxin‐2, BALF was collected and eosinophils were counted as Siglec F^+^/CD11c^−^ cells. (E) bmEos were isolated from WT or CB_2_‐KO mice, treated with JWH‐133 (250 nM) or vehicle, and were allowed to migrate toward serial dilutions of eotaxin‐2/CCL24. Data are shown as mean ± SEM, *P < 0.05, *n* = 4–7.

To explore the direct contribution of CB_2_ activation to eosinophil recruitment, *in vitro* and *in vivo* migration experiments were performed. As shown in Fig. 4A, JWH‐133 itself elicited a moderate chemotactic response in human eosinophils but not in mouse eosinophils (Fig. S2), similar to the previously noted effect of the endogenous CB_2_ agonist 2‐AG 19. The maximal chemotactic response (CI ~3.9) was observed at ~100 nM of JWH‐133. At micromolar levels JWH‐133 evoked chemokinesis, with a maximal response (CI ~1.8) observed at 3 μM. Moreover, at low nanomolar concentrations JWH‐133 (5 nM) significantly increased the migratory capacity of human eosinophils (Fig. 4B) and bmEos (Fig. 4C) toward eotaxin‐2/CCL24 by ~80% and 28%, respectively. Emphasizing the physiological importance of these observations, systemic administration of JWH‐133 (5 mg/kg/day) for three consecutive days significantly enhanced the eotaxin‐2/CCL24‐directed accumulation of eosinophils in the airways of IL‐5 transgenic mice by ~29% (Fig. 4D). To further confirm the direct involvement of CB_2_ in the recruitment of mouse eosinophils, bmEos were isolated from WT and CB_2_‐KO mice, treated with vehicle or JWH‐133 (250 nM), and then allowed to migrate toward serial dilutions of eotaxin‐2. Figure 4E shows that the priming effect of the cannabinoid agonist is not present in CB_2_‐KO mice, whereas JWH‐133 treatment resulted in a significantly increased migratory response of bmEos from WT mice.

### CB_2_ receptor activation enhances respiratory burst but not degranulation in human eosinophils

**Figure 5:**
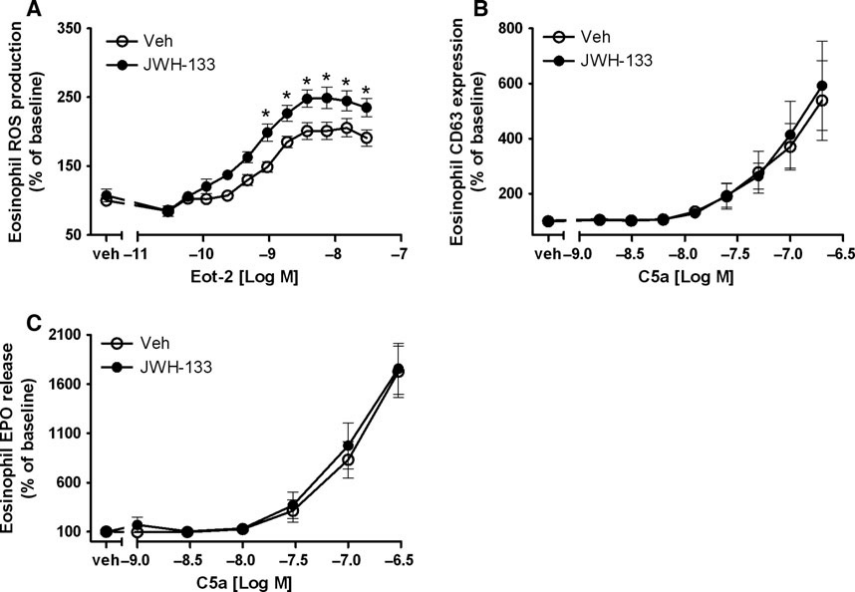
JWH‐133 enhances eosinophil respiratory burst but not degranulation. (A) Eosinophils were pretreated with JWH‐133 (250 nM) or vehicle, DCFDA (50 μM) was added, and cells were stimulated with eotaxin‐2/CCL24 for 30 min at 37°C. Data are shown as mean ± SEM,* n* = 6. (B and C) Eosinophils were pretreated with JWH‐133 (250 nM) or vehicle, and CD63 expression and EPO release were induced with serial dilutions of C5a. Data are shown as mean ± SEM,* n* = 5.

At sites of allergic inflammation, activated eosinophils cause tissue damage by the production of reactive oxygen species (ROS) and the release of toxic granule proteins 36. To explore the impact of selective CB_2_ activation on eosinophil respiratory burst, purified human eosinophils were applied to 250 nM JWH‐133 for 5 min at 37°C and respiratory burst was induced with eotaxin‐2/CCL24. As shown in Fig. 5A, JWH‐133 itself failed to induce respiratory burst, but significantly increased the eotaxin‐2/CCL24 induced ROS production up to ~25%.

Interestingly, although having significant effects on eosinophil recruitment and ROS production, JWH‐133 neither induced nor primed human eosinophil degranulation as assessed by means of CD63 upregulation and EPO release (Fig. 5B and C).

### JWH‐133 modulates eosinophil responses in a pertussis toxin (PTX)‐insensitive manner

**Figure 6:**
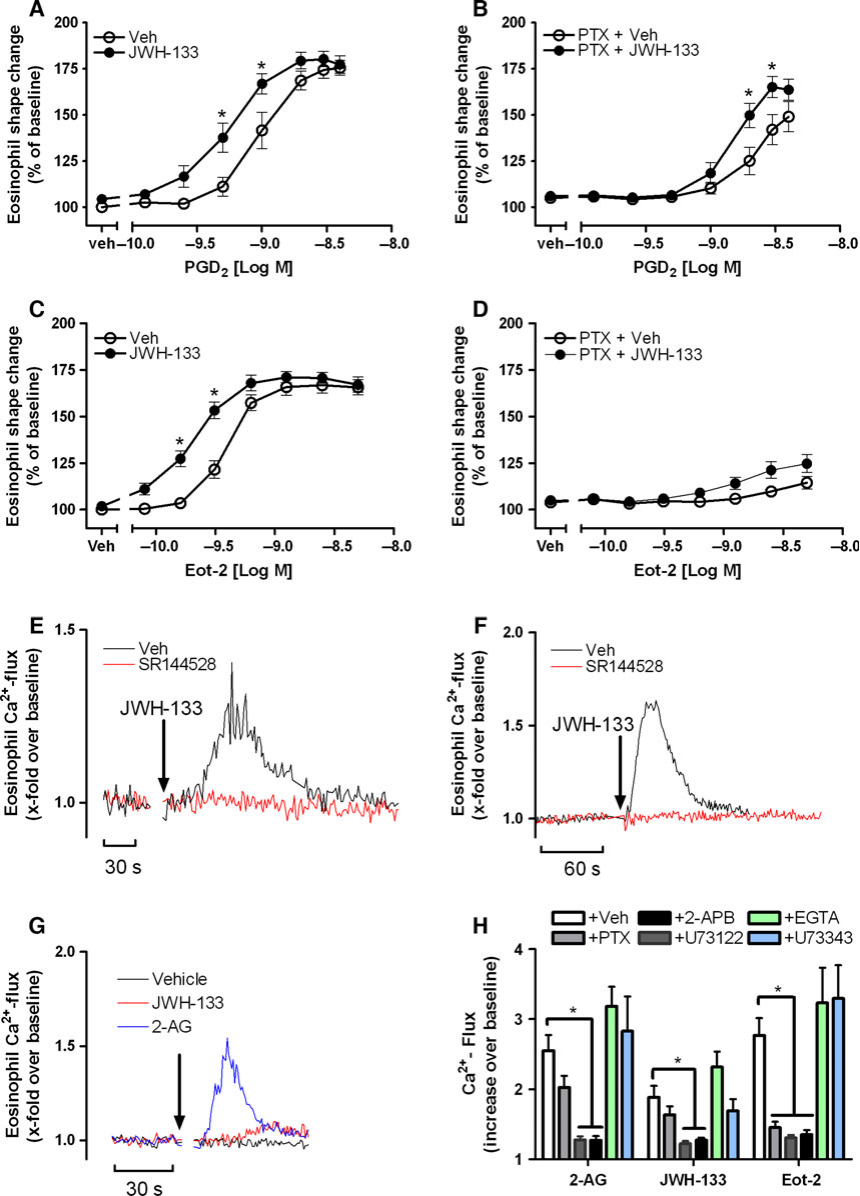
JWH‐133 modulates eosinophil function in a pertussis toxin (PTX)‐insensitive manner. Isolated human eosinophils were pretreated with vehicle (A and C) or PTX (5 μg/ml) (B and D) for 20 min at 37°C and exposed to JWH‐133 (100 nM) or vehicle for 5 min at room temperature. Shape change was stimulated for 4 min at 37°C with serial dilutions of PGD_2_ (A and B) or eotaxin‐2/CCL24 (C and D). Isolated human (E) or mouse (F) eosinophils were loaded with Fluo3‐AM and incubated with 1 μM SR144528 or vehicle, mixed with 200 nM JWH‐133, and Ca^2+^ flux was measured by FACS. G: Ca^2+^ mobilization was elicited by 100 nM of JWH‐133 and 2‐AG. Data are shown as representative of 3–5 independent experiments. (H) Eosinophil were pretreated with 5 μg/ml PTX for 20 min, with 6 μM U‐73122 or its inactive form U73343, 50 μM 2‐APB, or vehicle, and Ca^2+^ flux was induced by 200 nM JWH, 200 nM 2‐AG, or 1 nM eotaxin‐2/CCL24. In some experiments, EGTA (3 mM) was added prior to stimulation. Data are shown as mean ± SEM, *P < 0.05, *n* = 5–10.

CB_2_ receptors are known to activate heterotrimeric G_i/o_ type G proteins leading to the inhibition of adenylyl cyclase (AC). Furthermore, CB_2_ signaling was shown to involve MAPK (p38 and p42/44) and PI3K activity 37. To explore whether the observed effects depend on Gα_i_ activation, purified human eosinophils were incubated in the presence of pertussis toxin (PTX; 5 μg/ml for 20 min at 37°C) and shape change was induced with PGD_2_ (PTX‐insensitive) or eotaxin‐2/CCL24 (PTX‐sensitive). Notably, pretreatment with PTX did not affect the modulatory effects on PGD_2_‐induced shape change (Fig. 6A and B). As expected, PTX pretreatment almost completely blocked the Gα_i_‐dependent CCR3/CCL24 response but not the priming properties of JWH‐133 (Fig. 6C and D).

### JWH‐133 induces calcium release in human and mouse eosinophils

Having confirmed that CB_2_ signaling in human eosinophils is transmitted in a G_αi_‐independent manner, we next aimed to explore the role of Ca^2+^ in the CB_2_ pathway. To this end, responses of the endogenous ligand 2‐AG were compared with those of the synthetic agonist JWH‐133. At 100 nM, JWH‐133 only induced a weak Ca^2+^ signal in human eosinophils, whereas 2‐AG exhibited a robust fivefold stronger response (Fig. 6G). Consistent with JWH‐133 and 2‐AG sharing the same receptor, JWH‐133 (100 nM) desensitized the calcium response induced by 2‐AG (100 nM) (Fig. S3A). However, 100 nM of JWH‐133 or 2‐AG, concentrations that were effective in enhancing shape change responses in human eosinophils, did not increase eotaxin‐2/CCL24‐induced Ca^2+^ flux (Fig. S3B), neither did 200 nM of JWH‐133 (Fig. S3C), although at this concentration a robust and CB_2_‐dependent Ca^2+^ flux was found in human (Fig. 6E) and mouse eosinophils (Fig. 6F). To confirm the observed PTX insensibility of CB_2_ signaling, human eosinophils were treated with PTX (5 μg/ml, 20 min 37°C) and Ca^2+^ flux was induced with JWH‐133 (200 nM), 2‐AG (200 nM) or eotaxin‐2/CCL24 (1 nM), respectively. As expected, Ca^2+^ responses to eotaxin‐2/CCL24 were almost completely blocked, whereas JWH‐133‐ and 2‐AG‐induced Ca^2+^ responses remained unaffected (Fig. 6H). In contrast, pretreatment with the PLC inhibitor U‐73122 (but not its inactive form U73343), as well as the IP_3_ receptor antagonist 2‐APB abolished Ca^2+^ responses induced by JWH‐133 and 2‐AG indicating that CB_2_ interacts with Gα_q_ rather than Gα_i_. 2‐APB is also capable of inhibiting TRP channels. Thus, to exclude the involvement of TRP, control experiments in the presence of 3 mM EGTA were conducted, which showed that Ca^2+^ influx from the extracellular space and hence TRP channels are not involved in the Ca^2+^ response to CB_2_ receptor activation (Fig. 6H).

### Effects of JWH‐133 on human eosinophils are mediated via MEK1/2 and p160 ROCK

Human eosinophil migration is regulated by multiple signaling pathways involving PI3K, ROCK, ERK, and p38 MAPK 38. To assess the downstream components of the CB_2_ pathway in human eosinophils, cells were pretreated with LY‐294002 (PI3K, 10 μM), U‐0126 (MEK1/2, 10 μM), PD‐184161 (MEK1/2, 18 μM), SB202190 (p38 MAPK, 50 μM, Y‐27632 (ROCK, 200 nM) or vehicle, respectively. Concentrations were chosen according to the literature, and pretreatment was performed for 20 min at 37°C 38. Thereafter, eosinophil shape change was induced with eotaxin‐2/CCL24. As illustrated in Fig. S4, we found that the MEK1/2 inhibitors U‐0126 (B) and PD‐184161 (C) as well as the p160 ROCK inhibitor Y‐27632 (D) significantly reduced the priming effect of JWH‐133 for eotaxin‐2/CCL24 responses, whereas blockade of PI3K (E) and p38 MAP kinase (F) had no effect.

Having confirmed that JWH‐133 augments eosinophil responsiveness to other chemoattractants via MEK1/2 and ROCK signaling in shape change, we investigated the selective contribution of MEK1/2 and ROCK to JWH‐133‐induced chemotaxis. Similarly, pretreatment with U‐0126 and Y‐27632 prior to the chemotaxis assay effectively inhibited eosinophil migration toward serial dilutions of JWH‐133 (Fig. S4G and H).

### Systemic application of JWH‐133 worsens airway hyperreactivity (AHR) in mice

**Figure 7:**
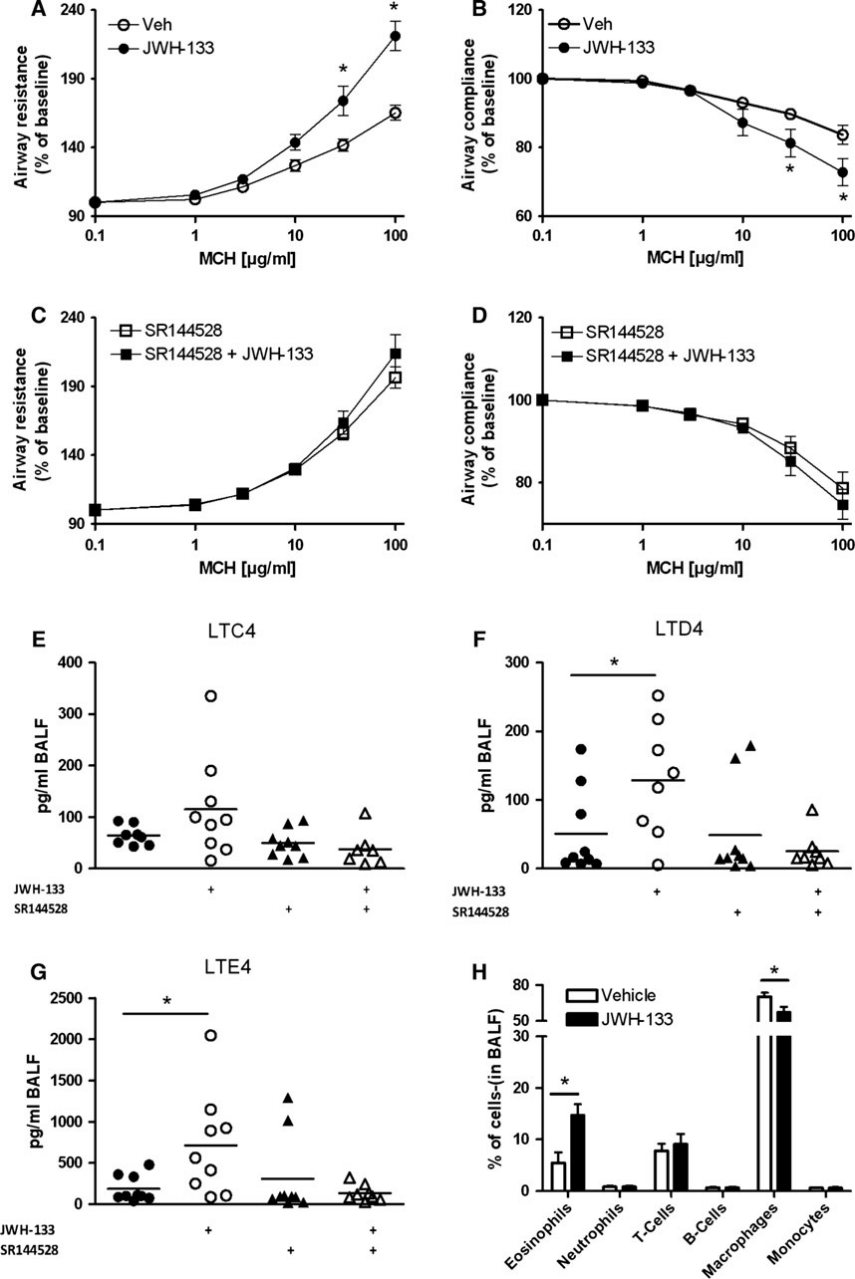
JWH‐133 deteriorates lung function and increases eosinophil counts in BALF. Eight‐week‐old female C57BL6 mice were sensitized and expose to ovalbumin and their lung function was assessed while applying increasing doses of methacholine (MCH) by a FlexiVent system. Mice were given either JWH‐133 (10 mg/kg) or vehicle from day 10 to 17 and airway resistance (A) and compliance (B) were measured. For (C and D), SR144528 (10 mg/kg) was applied alone or in combination with JWH‐133. (E–G) CysLT analysis in the BALF by mass spectrometry revealed slightly increased LTC4 but significantly enhanced LTD4 as well as LTE4 levels in the JWH‐133‐treated animals which could be reversed by SR144528. (H) Flow cytometric analysis of cells in the BALF of mice treated as in (A). Data are shown as mean ± SEM, * *P* < 0.05, *n* = 6–9.

Our *in vitro* data clearly show that CB_2_ ligands significantly contribute to eosinophil activation and responsiveness. To prove the *in vivo* relevance of these observations we used an acute model of OVA‐induced asthma in mice. In brief, mice were immunized to OVA on days 0 and 7, were treated i.p. with JWH‐133 (10 mg/kg), SR144528 (10 mg/kg), a combination of both, or vehicle from day 9 to day 16, and were challenged with inhaled OVA aerosol on days 14 and 16. On day 17, either airway hyperresponsiveness to methacholine was recorded, or BALF was taken. As illustrated in Fig. 7, JWH‐133 significantly impaired airway resistance and compliance compared to the vehicle group (Fig. 7A and B), whereas SR144528 pretreatment fully prevented these effects of JWH‐133 (Fig. 7C and D). Of the potent inflammatory lipid mediators comprising the cysteinyl leukotrienes (CysLTs: LTC_4_, LTD_4_, and LTE_4_), only LTE_4_ is stable and abundant *in vivo*. Besides mast cells, eosinophils are the main source of CysLTs which contribute not only to bronchoconstriction and airway hyperreactivity 39, but as in the case of LTE_4_ also to eosinophil recruitment 40. Increased levels of LTE_4_ can be detected in urine 41, BALF 42, and exhaled breath condensate 43 of patients after allergen challenge. Accordingly, mass spectrometric analysis of BALF from OVA‐challenged mice revealed slightly increased LTC4 levels (Fig. 7E) and significantly elevated concentrations of LTD_4_ and LTE_4_ in JWH‐133‐treated mice compared to vehicle‐treated controls. Again, the effect of JWH‐133 could be abolished with SR144528 pretreatment (Fig. 7F and G). We furthermore determined eosinophil counts in the BALF of JWH‐133‐ and vehicle‐treated mice. Consistent with our results from the *in vitro* and *in vivo* migration assays, flow cytometric analysis revealed threefold higher eosinophil counts in the JWH‐133‐treated group compared to controls (Fig. 7H). Representative histology images of lung sections are provided in Fig. S6. To confirm the eosinophil‐specific effect of the CB_2_ agonist to the pathogenesis of allergic asthma, further experiments in eosinophil‐deficient ∆dblGATA mice were conducted. Treatment with JWH‐133 worsened airway resistance in WT mice, but mediated beneficial effects on lung parameters in the ∆dblGATA group (Fig. S7A/B). Thus, our results show unequivocally that systemic CB_2_ activation directly contributes to the pathophysiology of asthma in mice by enhancing eosinophil migration and effector function.

## Discussion

In this study, we propose a novel mechanism of CB_2_‐induced priming of eosinophils that may directly contribute to the pathogenesis of eosinophilic diseases. This notion is supported by the observation that pretreatment with the selective and stable CB_2_ receptor agonist JWH‐133 profoundly increased eosinophil responsiveness toward chemoattractants such as eotaxin‐2/CCL24 and PGD_2,_ with respect to shape change, integrin expression, adhesion, chemotaxis and ROS production_._ The magnitude of primed responses was dependent on the concentration of JWH‐133 and of the chemoattractant. In line with these *in vitro* data, systemic application of JWH‐133 amplified the eotaxin‐2/CCL24‐directed recruitment of eosinophils into the airways of IL‐5Tg mice and exacerbated OVA‐induced asthmalike inflammation by increasing eosinophil influx into lungs and worsening of AHR. Oka et al. were the first who demonstrated that EoL‐1 cells and human peripheral blood eosinophils express CB_2_ but not CB_1_ receptors 30. Consistent with these data, we found that purified human eosinophils from healthy subjects express an appreciable amount of CB_2_ on mRNA (data not shown) and protein levels. Enhanced gene expression of CB_2_ has been shown for lung eosinophils in allergic patients after allergen challenge 44. Accordingly, we could demonstrate that also CB_2_ protein expression is significantly increased on the cell surface of peripheral blood eosinophils from symptomatic allergic donors, supporting a role for CB_2_ and its ligands in the regulation of allergen‐induced eosinophilic inflammation.

Activation with the selective agonist JWH‐133 at nanomolar levels only induced a slight chemotactic response in human eosinophils, but amplified eotaxin‐2/CCL24 induced eosinophil migration, especially at low concentrations. In contrast, IL‐8‐induced neutrophil activation remained unaffected by JWH‐133, which is in line with previous reports demonstrating that the effects of 2‐AG on neutrophils are mainly caused by the degradation of 2‐AG to AA and subsequent *de novo* synthesis of LTB_4_ 32.

Preactivation or priming of eosinophils by proinflammatory cytokines in the peripheral blood is a crucial step in the pathogenesis of allergic diseases. Several priming‐dependent eosinophil responses, such as migration 45, adhesion 46, and degranulation 47, have been shown to be amplified in allergic patients. Moreover, a ‘hyperadhesive’ eosinophil phenotype, characterized by increased levels of the adhesion molecule integrin α_M_β_2_ (CD11b/CD18) has been described in allergic patients after allergen challenge 48. Here we could show that exposure to the selective CB_2_ receptor agonist JWH‐133 prior to stimulation with chemoattractants yields significantly upregulated amounts of CD11b on the surface of both human and mouse eosinophils. Accordingly, JWH‐133 also primes eosinophils for an enhanced capacity to adhere to ICAM‐1 under physiological flow conditions, an important prerequisite of endothelial transmigration.

To further prove the *in vivo* relevance of the observed modulating activities of CB_2_, well‐established mouse models of directed eosinophil migration and OVA‐induced asthma were performed. Of note, we could show that daily treatment with JWH‐133 not only aggravated lung parameters, but also led to increased eosinophil counts in the airways of OVA‐challenged mice. Interestingly, these effects were completely absent in eosinophil‐deficient ∆dblGATA mice, indicating that eosinophils are the major target of JWH‐133 in allergic inflammation. Moreover, BALF of JWH‐133‐treated animals contained significantly higher CysLT levels compared to control animals; again, pointing to a more severe disease state due to systemic CB_2_ activation. Noteworthy, Larose et al. demonstrated that 2‐AG alone or in combination with platelet‐activating factor (PAF) induced CysLT biosynthesis in human eosinophils *in vitro*. However, 2‐AG/PAF‐induced synthesis was blocked by MAG lipase inhibitors, indicating that this effect is more related to 2‐AG degradation and metabolic transformation into eicosanoids than to selective CB_2_ activation 37. In contrast to 2‐AG, JWH‐133 is not degradable to AA, and therefore cannot be further used for CysLT synthesis indicating a direct correlation exists between the observed enhanced CysLT levels and activation of CB_2._ In accordance with our findings, recent work provided further evidence that CB_2_ receptor activation is capable of enhancing inflammatory processes. Treatment with the CB_2_ agonist JWH‐133 potentiated adipose tissue inflammation in mice on high fat diet 49. Notably, 2‐AG plasma levels were also found to positively correlate with the body mass index (BMI) in humans 50. Interestingly, the prevalence of asthma, its severity, and reduced responsiveness to standard medication seem to be associated with obesity and high BMI 51. However, the molecular mechanisms leading to these derangements are still poorly understood, but it is tempting to speculate that CB_2_ activation by systemically elevated 2‐AG levels and facilitation of eosinophil recruitment into the airways might be involved.

In both, human eosinophils and monocytes, 2‐AG‐induced CB_2_ signaling was reported to be Gα_i/o_‐dependent as pretreatment with PTX abrogated the chemotactic responses of these cells 30. Conversely, our data strongly suggest a Gα_i/o_/adenylyl cyclase‐independent pathway, substantiated by the fact that PTX was unable to prevent eosinophil priming and Ca^2+^ flux following CB_2_ activation. Furthermore, we observed that the PLC inhibitor U‐73122 and the IP_3_ receptor antagonist 2‐APB were capable of reducing JWH‐133‐ and 2‐AG‐induced Ca^2+^ release. Thus, eosinophil CB_2_ receptors seem to interact with Gα_q_ rather than, or in addition to, Gα_i_ proteins_._ Of note, Shoemaker et al. showed previously that cannabinoid agonists display different rank orders of potencies and receptor occupancies for regulation of intracellular effectors. Endogenous ligands such as 2‐AG are more ‘efficient’ agonists requiring only half the receptor occupancy to elicit same effects as synthetic agonists. Accordingly, we found that 2‐AG induced a much stronger Ca^2+^ response compared to JWH‐133 52. CB_2_ receptors have previously been shown to induce a G_βγ_‐dependent MAPK/ERK signaling cascade 53. Here we found that the induction of MEK 1/2 and ROCK activity is likely to be a part of the signaling mechanism accounting for the priming effect of JWH‐133. Our observations are in line with previous findings indicating the involvement of MEK1/2‐ERK activation in IL‐5 and GM‐CSF induced priming of human eosinophils 53. Moreover, MEK1/2‐ERK and ROCK signaling regulates a variety of proinflammatory cellular processes such as eosinophil migration, degranulation, and respiratory burst 38, 55 which afford the progression of eosinophilic inflammation.

In summary, the results of the present study demonstrate for the first time that specific CB_2_ activation represents a novel priming process leading to enhanced migratory responsiveness of human and mouse eosinophils *in vitro* and *in vivo*. This CB_2_‐mediated amplification of eosinophil migration seems to occur G_i/o_/adenylyl cyclase‐independent, but involves Gα_q_/MEK/ROCK signaling. Previous studies in patients with asthma showed a beneficial effect of inhaled or orally taken cannabinoids by dilating bronchial smooth muscles 56, 57. In contrast, our data provide evidence for the involvement of the endocannabinoid/CB_2_ axis in the progression of allergic inflammatory processes and indicate possible undesirable proinflammatory effects of long‐term cannabinoid use. Thus, specific CB_2_ receptor antagonism may open a new therapeutic approach for allergic disorders and other eosinophil‐driven diseases.

## Declaration of funding

E.S. received funding from the Franz Lanyar Stiftung of the Medical University of Graz (Grant No. 344) and was supported by the Jubilaeumsfonds of the Austrian National Bank (Grant No. 14446). A.F. was funded by the Karen and Sten Mörtstedt Initiative on Anaphylaxis. R.F. and M.P. were supported by the Austrian Science Fund FWF (DK‐MOLIN W1241), and A.H. was supported by the Austrian Science Fund FWF (Grant No. P22521) and Jubilaeumsfonds of the Austrian National Bank (Grant No. 14263).

## Conflicts of interest

The authors declare no conflict of interest.

## Supporting information


**Data S1** Supplemental Material.

**Figure S1** The endocannabinoid 2‐AG enhances eotaxin‐2/CCL24 induced CD11b upregulation.

**Figure S2** JWH‐133 does not induce chemotaxis in mouse eosinophils.

**Figure S3** CB_2_ desensitization and the effect on eotaxin‐2/CCL24 induced Ca^2+^ flux.

**Figure S4** MEK1/2 and p160 ROCK are involved in the modulating effect of JWH‐133.

**Figure S5** Representative images of eosinophil shape change assessed by flow cytometry.

**Figure S6** Representative histological pictures of paraffin sections of lungs from OVA‐challenged mice.

**Figure S7** Eosinophils are required for JWH‐133 induced aggravation of lung parameters.

**Table S1** Nomenclature for lipid mediators
**Table S2** Calibration levels of the lipid mediators (LTC_4_, LTD_4_ and LTE_4_) analyzed and concentration of internal standard ([^2^H_5_]‐LTD_4_, [^2^H_5_]‐LTC_4_, and [^2^H_5_]‐LTE_4_ ) after adding to the sample or to the calibration levels.
**Table S3** Analytical characterization of the lipid mediators´ standards.
