# Supplementary material for: Cannabinoid receptor 2 augments eosinophil responsiveness and aggravates allergen‐induced pulmonary inflammation in mice
Source: Allergy. 2016 Mar 16;71(7):944–56. doi: 10.1111/all.12858 (PMC5225803; doi:10.1111/all.12858)
Supplement: Supplementary file 1 — Data S1 Supplemental Material. [file ALL-71-944-s009.docx]

**Supplementary Figure Legends**

**Figure E1. The endocannabinoid 2-AG enhances eotaxin-2/CCL24 induced CD11b upregulation. A**: Human PMNL were stained with anti-CD11b-PE Ab and anti-CD16-PE-Cy5 Ab and were pretreated with 2-AG (250 nM) or vehicle and then stimulated with eotaxin-2/CCL24. Data are shown as mean ± SEM, n= 5.

**Figure E2. JWH-133 does not induce chemotaxis in mouse eosinophils.** BmEos were allowed to migrate towards serial dilutions of JWH-133 in a microBoyden chamber. Data are shown as mean ± SEM, n= 3.

**Figure E3. CB_2_ desensitization and the effect on eotaxin-2/CCL24 induced Ca^2+^ flux.**

**A**: Stimulation with JWH-133 (100 nM) desensitizes the CB_2_ receptor for further 2-AG (100 nM) responses. **B:** Pre-treatment with 100 nM JWH-133 or 100 nM 2-AG does not affect responses induced by eotaxin-2/CCL24 (1.5 nM). Data are shown as a representative of 3-5 independent experiments. **C:** Pre-treatment with 200 nM JWH-133 does not affect eotaxin-2/CCL24 induced responses. Data are shown as mean ± SEM, n= 5.

**Figure E4. MEK1/2 and p160 ROCK are involved in the modulating effect of JWH-133.** Eosinophils were pre-treated with the protein kinase inhibitors LY-294002 (PI3K, 10 µM, **B**), U-0126 (MEK1/2, 10 µM, **C**), PD-184161 (MEK1/2, 18 µM, **D**), SB202190 (p38 MAPK, 50 µM, **E**) and Y-27632 (ROCK, 200 nM, **F**) or vehicle (**A**). Following incubation with JWH-133 (100 nM) or vehicle, eosinophils were stimulated with eotaxin-2/CCL24. In **G** and **H** eosinophils were pretreated with U-0126 and Y-27632 or vehicle and were allowed to migrate towards serial dilutions of JWH-133. Data are shown as mean ± SEM, * p<0.05, n= 5-14.

**Figure E5. Representative images of eosinophil shape change assessed by flow cytometry. A:** FSC/SSC plot of unstimulated isolated human eosinophils. **B:** FSC/SSC plot of isolated human eosinophils stimulated with 0.6 nM eotaxin-2/CCL24. Due to the rearrangement of the cytoskeleton upon activation, the cell size increases, which is detectable as an increase of the FSC. **C:** Same as in **B**, but eosinophils were pretreated with 100 nM JWH-133, leading to a further increase of the FSC parameter.

**Figure E6. Representative histological pictures of paraffin sections of lungs from OVA-challenged mice.** Mice were treated as in **Fig. 7A** and lung sections were fixed with 4% formaldehyde and embedded in paraffin. Lung histopathology was evaluated by H&E and visually examined with an Olympus IX70 microscope; photographs were taken with an Olympus DP50 camera. **A:** vehicle treated mice. **B**: JWH-133 treated mice.

**Figure E7. Eosinophils are required for JWH-133 induced aggravation of lung parameters.** Mice were treated as in (**Fig 7A**). **A:** BALB/c WT mice. **B:** ∆dblGATA mice. Data are shown as mean ± SEM, * p< 0.05, n=3 - 6.

**Supplemental Material**

**Materials**

All reagents were from Sigma (Vienna, Austria), unless speciﬁed. Assay buffer was made from Dulbecco`s modiﬁed PBS (with 0.9 mmol/L Ca^2+^ and 0.5 mmol/L Mg^2+^; Invitrogen, Vienna, Austria), 0.1% BSA, 10 mmol/L HEPES, and 10 mmol/L glucose, pH 7.4. Human eotaxin-2/CCL24 was from R&D Systems (Minneapolis, USA). IL-8 was from Peprotech (London, United Kingdom). PGD_2_, SR144528, JWH-133, 2-APB, U-73122, U-73343 and 2-AG were purchased from Tocris (Bristol, UK). Anti-CD16-PE-Cy5, anti-HLA-DR-FITC, anti-CD123-PE and anti-CD11b-PE (ICRF44) were from Becton Dickinson (Vienna, Austria). Polyclonal rabbit anti-human CB_2_ Ab was from Abcam (Cambridge, United Kingdom) and rabbit IgG isotype control was supplied from Santa Cruz Biotechnology (Heidelberg, Germany). The PI3K inhibitor LY294002 was supplied from Biomol (Hamburg, Germany). The MEK1/2 inhibitor U-0126, the p38 MAPK inhibitor SB 202190 and the ROCK inhibitor Y-27632 were purchased from Tocris (Bristol, UK). The MEK1/2 inhibitor PD 184161 was from Cayman Chemicals (Ann Abor, MI). CellFix and FACS-Flow were from Becton Dickinson (Vienna, Austria). PVP-free polycarbonate filters were from NeuroProbe (Gaithersburg, USA) and 96-well chemotaxis plates were from Corning Life Sciences (Radnor, USA). Fluo-3-AM was supplied from Life Sciences (Vienna, Austria).

Fixative solution was prepared by adding 9 ml of distilled water and 30 ml of FACS-Flow to 1 ml of CellFix.

**Blood donors:**

Blood was collected in- and off-season from healthy and allergic volunteers according to a protocol approved by the Ethics Committee of the Medical University of Graz. Medical history and allergic symptoms were assessed using a standardized questionnaire asking for physician-diagnosed allergies and the presence, nature and frequency of allergic symptoms. In this study, only healthy non-allergic subjects and subjects allergic to aeroallergens with respiratory symptoms were included.

**Mouse strains:**

Female C57Bl6/N mice were obtained from Charles River (Sulzfeld, Germany). Breeding pairs of the interleukin-5 transgenic (IL-5Tg) and eosinophil-deficient *Δdbl*GATA strains (BALB/c background) were a kind gift of Dr. Helene Rosenberg and maintained on-site. Mice were housed in individually ventilated cages (5 per cage) under controlled conditions of temperature (set point 21 °C), air humidity (set point 50%) and a 12 h light/dark cycle (lights on at 6:00 a.m.). Standard chow (altromin 1324 FORTI, Altromin, Lage, Germany) and water were provided ad libitum. The experimental procedure used in this study was approved by the Austrian Federal Ministry of Science, Research and Economy (protocol numbers: BMWF-66.010/0094-II/3b/2013; BMWFW-66.010/0020-WF/V/3b/2015) conform to Directive 2010/63/EU, and was performed in accordance with national and international guidelines. Mice were randomly assigned before treatment.

**Methods**

**Preparation of human basophils and eosinophils:**

Basophils were identified as CD123^+^/HLA-DR^-^ cells in PBMC preparations and eosinophils were isolated from PMNL by negative magnetic selection as described previously (56,57). Purity of the isolated eosinophils was typically greater than 97%.

**Preparation of mouse eosinophils:**

Bone marrow-derived eosinophils (bmEos) were differentiated *ex vivo* from unselected bone marrow progenitors using a well-defined cytokine regimen (25). Our cultures yielded high-purity (90-100%) bmEos by day 10-12 of culture as estimated by visual inspection of Diff-Quik stained cytospin slides.

**Flow cytometric staining of CB_2_ receptors on eosinophils**

Purified human eosinophils were stained with a polyclonal rabbit anti-human CB_2_ primary Ab or isotype control, followed by a goat anti-rabbit secondary Ab (AF-647). CB_2_ expression was quantified by flow cytometry.

**Shape change assay:**

Isolated eosinophils, PMNL or PBMC were pre-treated as indicated and stimulated with serial dilutions of agonists for 4 min at 37 °C. Shape change was estimated by flow cytometry as the increase of forward scatter and was expressed as percent of the vehicle response. Neutrophils were identified as CD16^+^ cells in PMNL preparations. Basophils were identified as CD123^+^/HLA-DR^-^ cells in PBMC preparations.

**Migration:**

Chemotaxis: Purified human eosinophils or bmEos were pre-treated as indicated, placed into the top of a 48-well micro-Boyden chamber (human) or a 96-well chemotaxis plate (bmEos) and were allowed to migrate towards the indicated chemoattractant for 1 hour (human) or 3 hours (bmEos) at 37 °C (25,26). Chemokinesis: human eosinophils were pre-treated as indicated and were allowed to migrate towards assay buffer. Migrated cells in the bottom wells were enumerated by flow cytometry.

**Calcium flux:**

Isolated human eosinophils or bmEos (10^7^cells/ml) were treated with 2 µM of Fluo-3-AM in the presence of 0.02% pluronic F-127 (26). Changes in intracellular Ca^2+^ were detected as the increase of the fluorescence of the Ca^2+^ sensitive dye Fluo-3 in the FL1-(530/30nm) channel.

**Adhesion assay under flow:**

Vena8^TM^ biochips (Cellix Ltd., Dublin, Ireland) were coated with 10 µg/ml ICAM-1 and coated channels were superfused with 2x 10^6^ purified eosinophils at 0.5 dyne/cm^2^ for 5 min at 37 °C in a heated cage. Adhesion was monitored using a Hamamatsu ORCA-03G digital camera and CellixVenaFlux software. Cell adhesion was quantiﬁed by computerized image analysis using DucoCell analysis software (Cellix Ltd, Dublin, Ireland).

**CD11b-upregulation:**

Whole blood samples or PMNL were pre-treated as indicated and incubated with agonists for 30 min at 37 °C (27). Samples were stained with anti-CD16-PE-Cy5 and anti-CD11b-PE (ICRF44) Ab. CD11b upregulation was analyzed by flow cytometry and expressed as percent of the vehicle response. Eosinophils were identified as CD16^-^ cells in the granulocyte forward scatter /side scatter region.

**Respiratory burst:**

Purified human eosinophils were pre-treated as indicated. Cells were stimulated with serial dilutions of eotaxin-2/CCL24 in the presence of 50 µM DCFDA for 20 min at 37 °C. Respiratory burst was immediately quantified by flow cytometry as the increase in the FL-1 channel due to the conversion of DCFDA into DCF by reactive oxygen. Responses were expressed as percent of the vehicle response.

**CD63 expression:**

Purified human eosinophils were pre-treated for 5 min at RT as indicated and labelled with anti-CD63-FITC Ab (26). Samples were treated with cytochalasin B (5 µg/ml) for 5 min at 37 °C and degranulation was induced with serial dilutions of complement component 5a (C5a) for 20 min at 37 °C. CD63 expression was detected by flow cytometry and expressed as percent of the respective control responses.

**EPO release:**

Purified eosinophils were treated with cytochalasin B (10 µg/ml) and aliquots were transferred into the wells of a 96-well microplate (58). After stimulation with serial dilutions of C5a for 20 min at 37 °C, H_2_O_2_ (1 mM) was added to start the peroxidase reaction. Tetramethylbenzidine (2.8 mM) was added to detect the reaction and colour development was terminated by addition of 4 M acetic acid. EPO release was quantified on a bench reader at a wavelength of 630 nm.

***In vivo* chemotaxis:**

Eight week-old IL-5Tg mice were treated i.p. with JWH-133 (5 mg/kg/d) or vehicle for three consecutive days. *In vivo* chemotaxis of eosinophils was induced by intranasal instillation of 4µg eotaxin-2/CCL24. Bronchoalveolar lavage fluid (BALF) was collected 5 h post-instillation, and migration was evaluated by flow cytometric counting of highly granular (high side scatter) CD11c^-^/Siglec F^+^ cells.

**Mouse model of allergic lung inflammation:**

Eight week old female C57Bl6/N mice were immunized by i.p. injections of 10 µg of OVA adsorbed to Al(OH)_3_ on days 0 and 7. Mice were challenged by an aerosol of 0.5% (w/v) of OVA in saline for 30 min on days 14 and 16. Additionally, mice received a daily i.p. injection of CB_2_ agonist/antagonist (10 mg/kg) or vehicle on day 9 to day 16. On day 17 either airway hyperresponsiveness to methacholine was recorded with the FlexiVent system (Scireq, Montreal,CA) or BALF was taken using 3ml PBS/EDTA (1mM), and analyzed by flow cytometry. Eosinophils in BALF were identified as highly granular (high side scatter) CD11c^-^/Siglec F^+^ cells, T-Cells as CD3e^+^, B-Cells as I-A/I-E^+^/B220^+^, macrophages as CD11c^+^/Siglec F^+^, monocytes as I-A/I-E^+^/CD11c^-^ and neutrophils as Siglec F^-^ cells in the granulocyte region. For histological analysis, lung tissue was fixed in 4% formaldehyde and embedded in paraffin. Ten-µm sections were stained with H&E and pictures were taken at a 100x magnification on an Olympus IX70 microscope with an Olympus DP50 camera.

**LC-MS analysis of BALF metabolite:**

A stable isotope dilution liquid chromatography-mass spectrometry (LC-MS) method was used, previously reported elsewhere (59), with slight modification to quantify cysteinyl leukotrienes (CysLTs) LTC4, LTD4 and LTE4. The complete method is described in the Supplemental Information, with lipid mediator nomenclature provided in Table E1.

**Statistical analysis:**

Data are shown as mean ± SEM for n observations, where n denotes independent experiments with cells from different donors. Comparisons of groups were performed using one-way ANOVA followed by Newman-Keuls´ post-hoc test or two-way ANOVA for repeated measurements followed by Bonferroni’s post-hoc test to determine the levels of significance for each group. Probability values of p < 0.05 were considered as statistically significant.

**LC-MS measurement:**

*SPE extraction*

Extraction of analytes was carried out in 3cc/60 mg HLB Oasis SPE cartridges (Waters). Cartridges were conditioned with 2 mL of methanol followed by 2 mL of water. A cocktail of deuterated internal standards (10 µL; Table E2) was added to 800 to 900 µL BALF sample, followed by 10 µL BHT/EDTA solution (0.2 mg/ml, dissolved in methanol/water 50:50, v/v) and 900 µL extraction buffer (citric acid: 0.2 M Na_2_HPO_4_, pH5.6) and loaded onto the SPE cartridge. Samples were washed with 2 mL of water/methanol (80:20, v/v) and the cartridges were dried in the manifold under vacuum-induced air stream at −30 kPa for 30 min. Analytes were eluted from the columns with 2.5 mL of methanol in cryotubes containing 10 µL of glycerol 30% in methanol. After evaporation of the eluates under vacuum, samples were resuspended in 60 µL of methanol. Additionally 10 µl of water was added to the samples. Solution was filtered by centrifugation in Amicon Ultrafree-MC, PVDF 0.1 µm (Millipore) and transferred to autosampler vials with inserts before injection.

*UHLC-MS/MS measurement*

Liquid chromatography coupled to mass spectrometry (LC-MS/MS) separation and quantification was carried out on a UPLC Acquity-Xevo TQS mass spectrometer system (Waters, Milford, MA). The autosampler and column were kept at 5°C and 60°C, respectively. Injection volume was set to 7.5 μL. Separation was achieved with an Acquity UPLC BEH C18 (2.1 × 150 mm, 1.7 μm, Waters).

For negative electrospray ionization (ESI) a gradient of solvents A (water with 0.1% of acetic acid) and B (acetonitrile/isopropanol 90:10, v/v) at a flow of 0.5 mL min^−1^ was used. The gradient initiated with 80% of A, which was decreased linearly to 65% at 2.5 min, to 60% at 4.5 min, to 58% at 6 min, to 50% at 8 min, to 35% at 14 min, to 27.5% at 15.50 min and to 0% at 16.60min. The column was then washed with solvent B for 0.9 min and equilibrated to initial conditions. For positive ESI a gradient of solvents A (water with 0.2% of formic acid) and B (acetonitrile/isopropanol 90:10, v/v + 0.2% formic acid) at a flow of 0.45 mL min^−1^ was used. The gradient initiated with 60% of A, which was decreased linearly to 50% at 4.25 min and to 5% at 4.5 min. The column was then washed with 95 % of solvent B for 3 min and equilibrated to initial conditions.

Data was collected on a Waters Xevo TQS-MS mass spectrometer using positive and negative electrospray ionization (ESI) and scheduled multiple reaction monitoring (MRM) mode. Dwell time was automatically adjusted in order to acquire 20 points per chromatographic peak; capillary voltage was 2.20 kV in positive and negative ESI. Desolvation temperature and gas flow were set according to the instrument recommendations for the chromatographic flow. Detailed MRM transition and chromatographic retention time for each compound is provided in Table E3.
