# Supplementary figures and images for: Cannabinoid receptor 2 augments eosinophil responsiveness and aggravates allergen‐induced pulmonary inflammation in mice
Source: Allergy. 2016 Mar 16;71(7):944–56. doi: 10.1111/all.12858 (PMC5225803; doi:10.1111/all.12858)

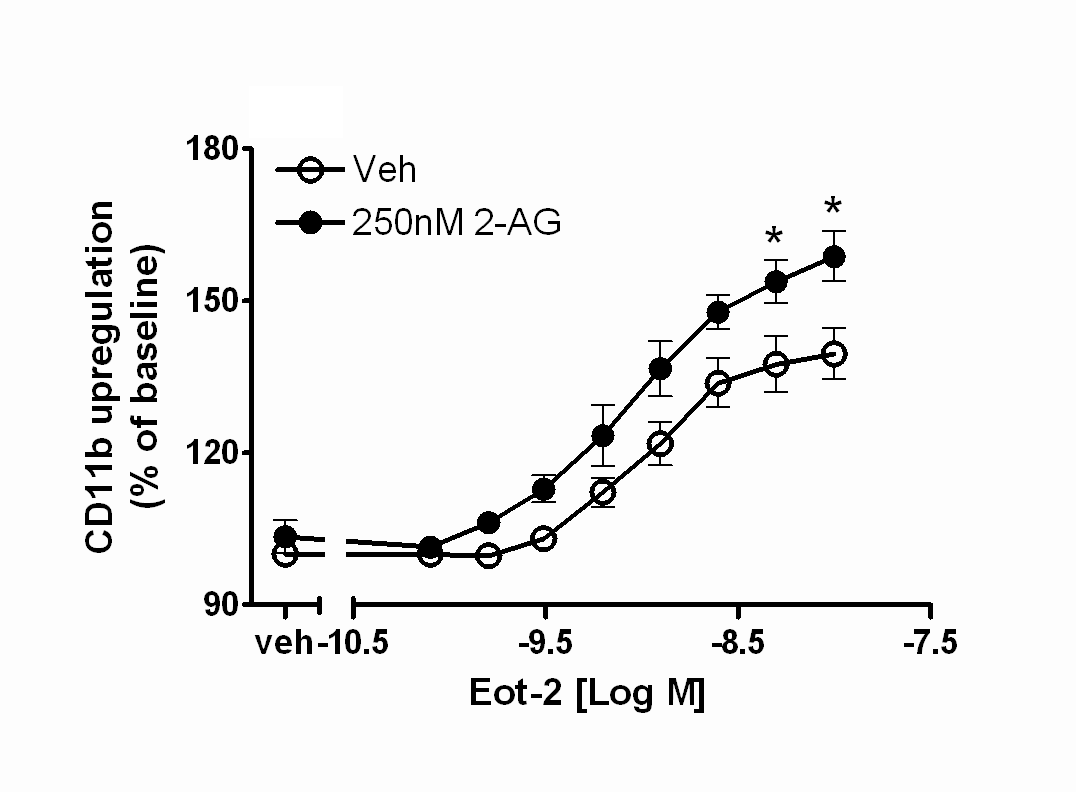

Supplement: Supplementary file 2 — Figure S1 The endocannabinoid 2‐AG enhances eotaxin‐2/CCL24 induced CD11b upregulation. [file ALL-71-944-s001.tif]

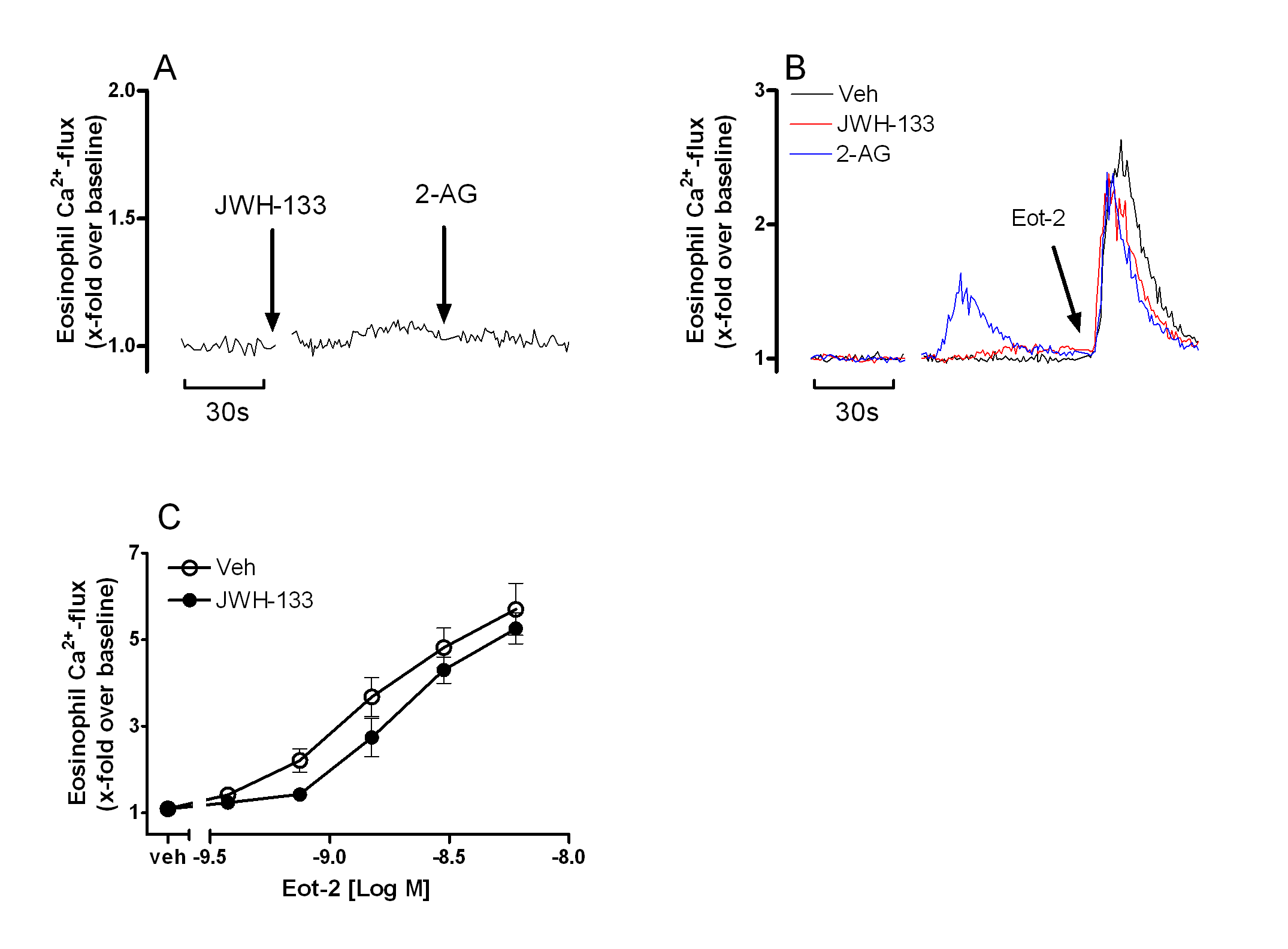

Supplement: Supplementary file 4 — Figure S3 CB2 desensitization and the effect on eotaxin‐2/CCL24 induced Ca2+ flux. [file ALL-71-944-s003.tif]

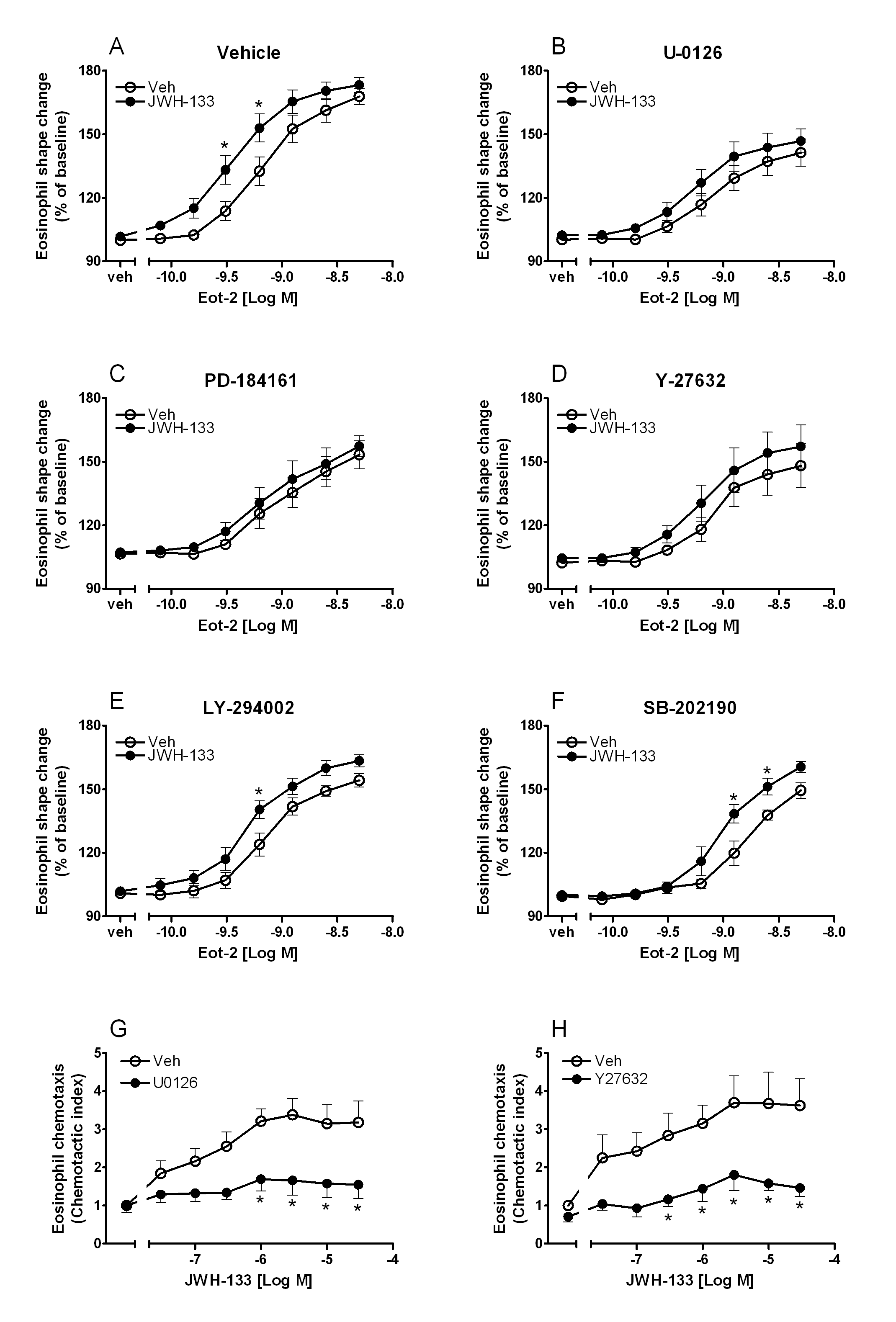

Supplement: Supplementary file 5 — Figure S4 MEK1/2 and p160 ROCK are involved in the modulating effect of JWH‐133. [file ALL-71-944-s004.tif]

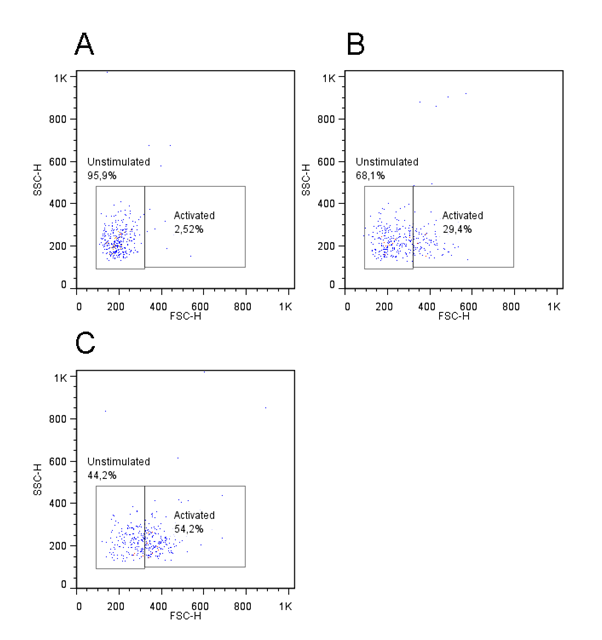

Supplement: Supplementary file 6 — Figure S5 Representative images of eosinophil shape change assessed by flow cytometry. [file ALL-71-944-s005.tif]

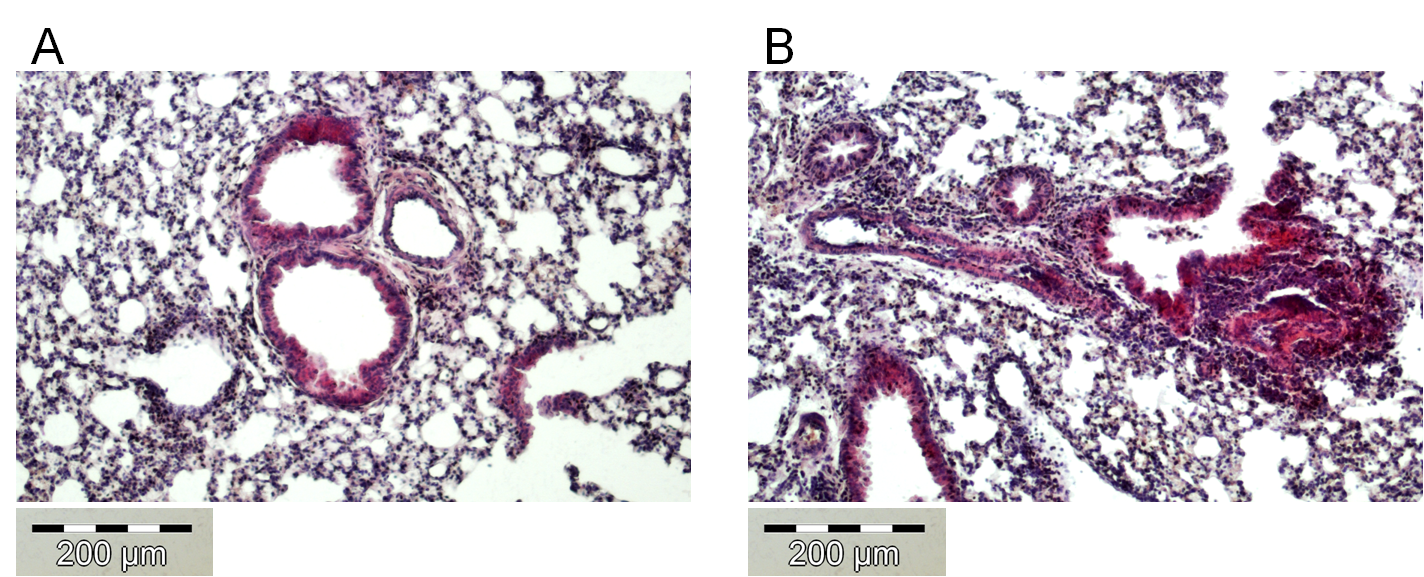

Supplement: Supplementary file 7 — Figure S6 Representative histological pictures of paraffin sections of lungs from OVA‐challenged mice. [file ALL-71-944-s006.tif]

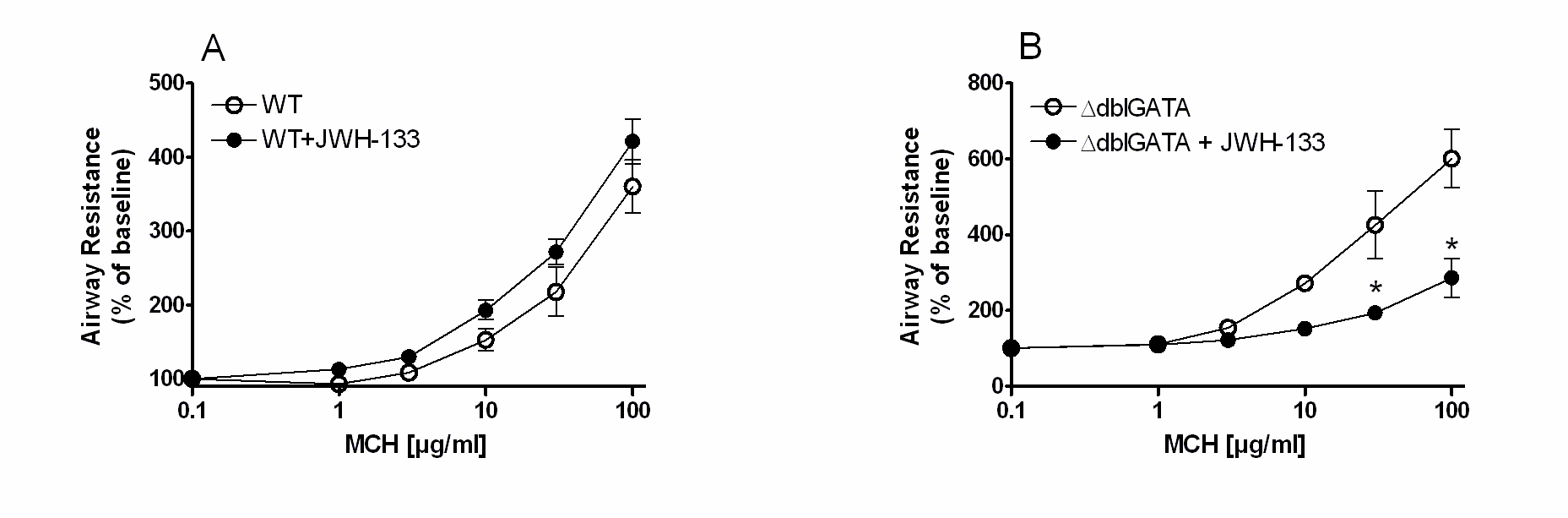

Supplement: Supplementary file 8 — Figure S7 Eosinophils are required for JWH‐133 induced aggravation of lung parameters. [file ALL-71-944-s007.tif]
