# Supplementary material for: Cannabinoid receptor 2 augments eosinophil responsiveness and aggravates allergen‐induced pulmonary inflammation in mice
Source: Allergy. 2016 Mar 16;71(7):944–56. doi: 10.1111/all.12858 (PMC5225803; doi:10.1111/all.12858)
Supplement: Supplementary file 9 — Table S1 Nomenclature for lipid mediators Table S2 Calibration levels of the lipid mediators (LTC4, LTD4 and LTE4) analyzed and concentration of internal standard ([2H5]‐LTD4, [2H5]‐LTC4, and [2H5]‐LTE4 ) after adding to the sample or to the calibration levels. Table S3 Analytical characterization of the lipid mediators´ standards. [file ALL-71-944-s008.docx]

**Supplemental tables LC-MS measurement:**

**Table E1.** Nomenclature for lipid mediators

|  |  |  |
| --- | --- | --- |
| **Abbreviation** | **Common name** | **Systematic name** |
|  |  |  |
|  |  |  |
| LTC_4_ | leukotriene C4 | 5S-​hydroxy-​6R-​(S-​glutathionyl)-​7E,​9E,​11Z,​14Z-​eicosatetraenoic acid |
| LTD_4_ | leukotriene D4 | 5S-​hydroxy-​6R-​(S-​cysteinylglycinyl)-​7E,​9E,​11Z,​14Z-​eicosatetraenoic acid |
| LTE_4_ | leukotriene E4 | 5S-​hydroxy-​6R-​(S-​cysteinyl)-​7E,​9E,​11Z,​14Z-​eicosatetraenoic acid |
|  |  |  |

**Table E2.** Calibration levels of the lipid mediators (LTC_4_, LTD_4_ and LTE_4_) analyzed and concentration of internal standard ([^2^H_5_]-LTD_4_, [^2^H_5_]-LTC_4_, and [^2^H_5_]-LTE_4_ ) after adding to the sample or to the calibration levels.

|  |  |  |  |  |  |  |  |  |  |  |  |
| --- | --- | --- | --- | --- | --- | --- | --- | --- | --- | --- | --- |
|  | Calibration level concentration (ng/ml) | | | | | | | | | | |
|  | 11 | 10 | 9 | 8 | 7 | 6 | 5 | 4 | 3 | 2 | 1 |
|  |  |  |  |  |  |  |  |  |  |  |  |
| LTC_4_ | 185.71 | 58.57 | 14.29 | 4.29 | 1.26 | 0.63 | 0.32 | 0.16 | 0.08 | 0.04 | 0.02 |
| LTD_4_ |  |  |  |  |  |  |  |  |  |  |  |
| LTE_4_ |  |  |  |  |  |  |  |  |  |  |  |
| [^2^H_5_]-LTC_4_ | 18.29 | | | | | | | | | | |
| [^2^H_5_]-LTD_4_ | 18.29 | | | | | | | | | | |
| [^2^H_5_]-LTE_4_ | 22.86 | | | | | | | | | | |
|  |  |  |  |  |  |  |  |  |  |  |  |

**Table E3.** Analytical characterization of the lipid mediators´ standards. **Ionization mode,** Chromatographic retention time (RT), parent ion, product ion, the internal standard (IS) assigned to every lipid mediator and the on-column injected linear range are reported.

|  |  |  |  |  |  |  |
| --- | --- | --- | --- | --- | --- | --- |
| Lipid mediator | ionization mode | RT | Parent ion | Product ion | IS | Linear range (fmol) |
|  |  | (min) | (m/z) | (m/z) |  |  |
|  |  |  |  |  |  |  |
|  |  |  |  |  |  |  |
| LTC_4_ | [M+H]^+^ | 3.61 | 626.2 | 308.2 | [^2^H_5_]-LTC_4_ | 0.2-2226 |
| LTD_4_ | [M+H]^+^ | 3.46 | 497.1 | 189.2 | [^2^H_5_]-LTD_4_ | 0.3-2804 |
| LTE_4_ | [M-H]^-^ | 7.6 | 438.4 | 333.3 | [^2^H_5_]-LTE_4_ | 0.3-3169 |
| [^2^H_5_]-LTC_4_ | [M+H]^+^ | 3.58 | 631.1 | 308.2 |  | |
| [^2^H_5_]-LTD_4_ | [M+H]^+^ | 3.43 | 502.2 | 194.2 |  | |
| [^2^H_5_]-LTE_4_ | [M-H]^-^ | 7.55 | 443.1 | 338.1 |  | |
|  |  |  |  |  |  | |
